# Supplementary material for: Heart-Rate-to-Blood-Pressure Ratios Correlate with Malignant Brain Edema and One-Month Death in Large Hemispheric Infarction: A Cohort Study
Source: Diagnostics (Basel). 2023 Jul 27;13(15):2506. doi: 10.3390/diagnostics13152506 (PMC10416946; doi:10.3390/diagnostics13152506)
Supplement: Supplementary file 1 [file diagnostics-13-02506-s001.zip › Supplementary Materials.pdf]

Research Article

Heart Rate-to-Blood Pressure Ratios Correlate with Malignant Brain Edema and One-month Death in Large Hemispheric Infarction: A Cohort Study

Xindi Song[a], Yanan Wang[a], Wen Guo[a], Meng Liu[a], Yilun Deng[a], Kaili Ye[a], Ming Liu[a]

[a] Department of Neurology, West China Hospital, Sichuan University, Chengdu, China

Short Title: Heart Rate-to-Blood Pressure Ratios Correlate with Malignant Brain Edema

Corresponding Author:

Ming Liu

Department of Neurology,

West China Hospital, Sichuan University

37 Guo Xue Xiang

Chengdu, 610041, China

Tel: 18980601671

E-mail: wyplmh@hotmail.com

Keywords: malignant brain edema; shock index; blood pressure; heart rate; biomarkers

Figure S1. The flow diagram of patient selection LHI, large hemispheric infarction; PH, parenchymal hemorrhage; BP, blood pressure; HR, heart rate

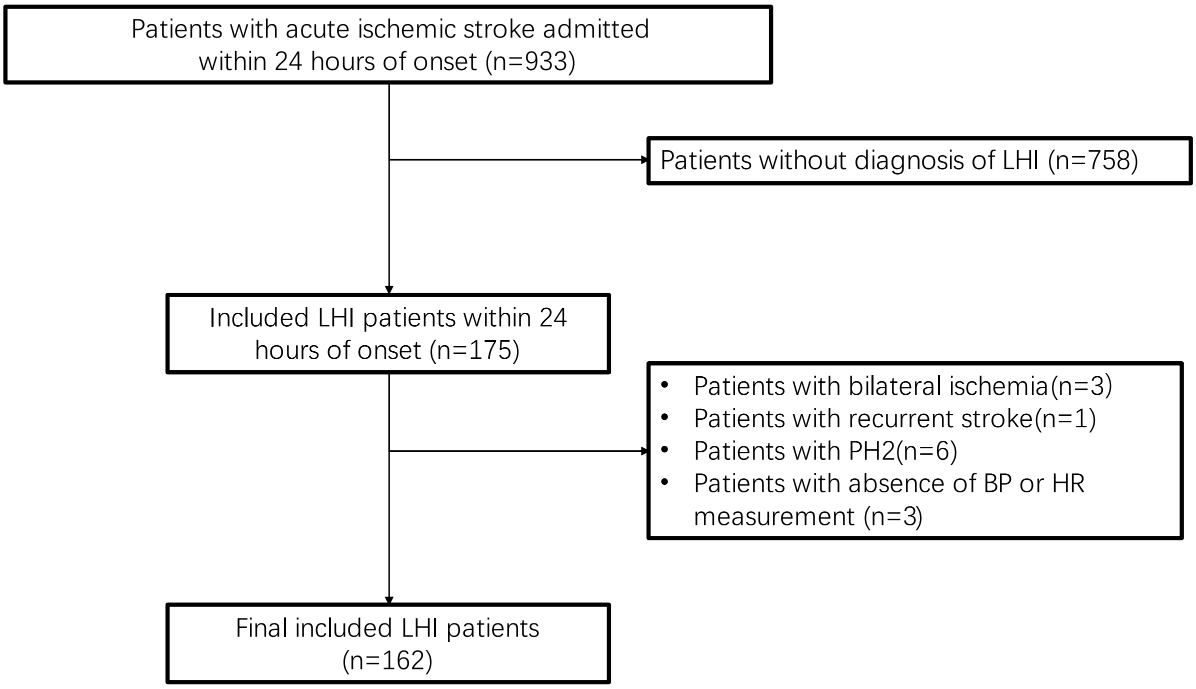

Figure S2. The 24-hour temporal evolution of heart rate/blood pressure ratios in LHI patients who died and survived within one month. (a)Temporal evolution of SI_s. (b)Temporal evolution of SI_d. SI, shock index; LHI, large hemispheric infarction; Heart rate-to-blood pressure ratios are assigned as SI_s and SI_d for systolic and diastolic pressures, respectively.

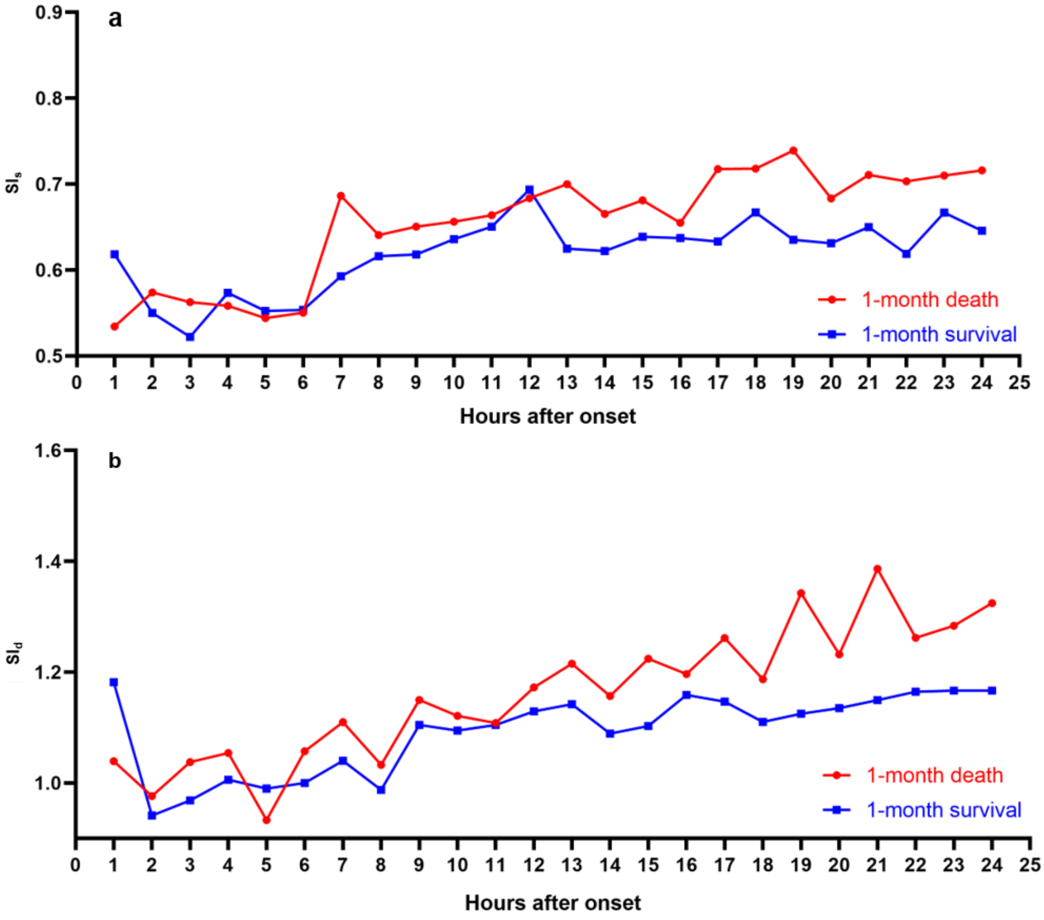

Figure S3. Kaplan-Meier survival curves for cumulative incidence of one-month death among LHI patients with high or low heart rate/blood pressure ratios during 13-24h after onset. Patients were categorized by median of SI_s and SI_d during 13-24h after onset, respectively. (a)Patients with higher SI_s had higher cumulative incidence of one-month death (log-rank P=0.010). (b) Patients with higher SI_d had higher cumulative incidence of one-month death (log-rank P < 0.001). LHI, large hemispheric infarction; SI, shock index; Heart rate-to-blood pressure ratios are assigned as SI_s and SI_d for systolic and diastolic pressures, respectively;\.

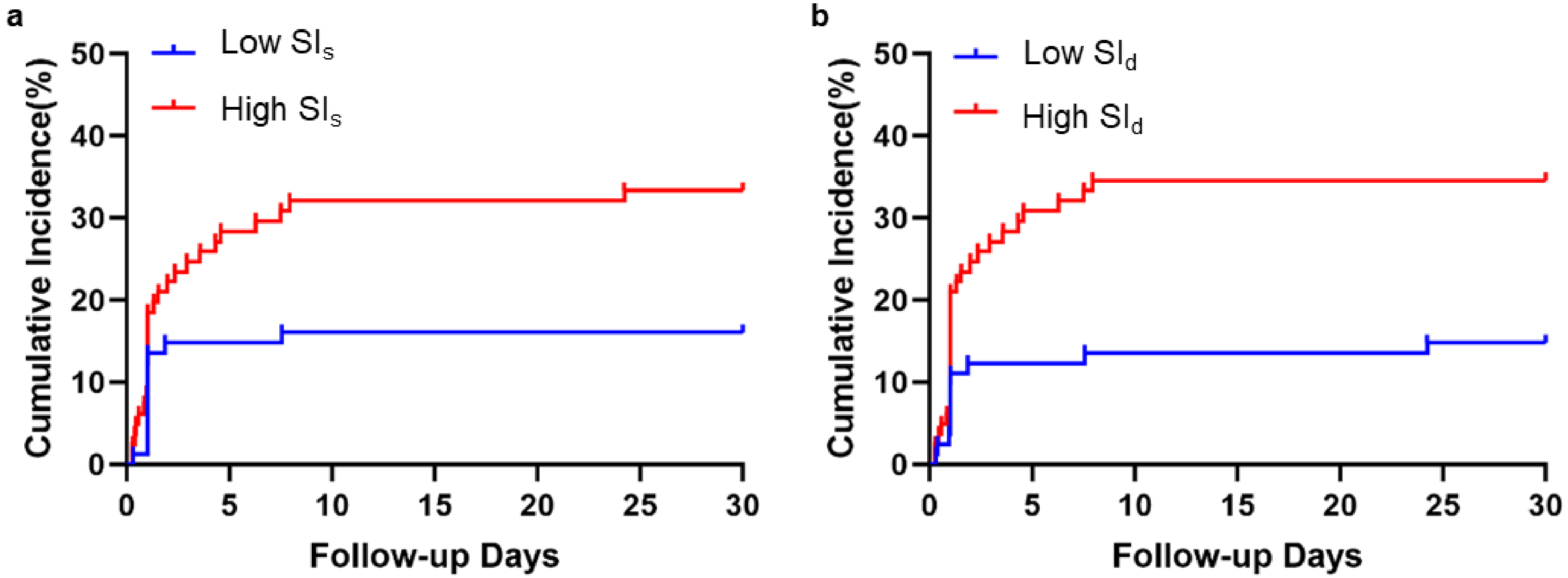

Table S1 The baseline characteristics of large hemispheric infarction patients who died and survived within one month

	Total (n=162)	One month death(n=40)	One month survival(n=122)	P values
**Demographics**				
Age,years(mean±SD)	70.31±11.76	71.00±10.69	70.08±12.13	0.670
Male,n(%)	71(43.83)	21 (52.50)	50 (40.98)	0.203
**Medical history**				
Hypertension,n(%)	79(48.77)	20 (50.00)	59 (48.36)	0.857
DM,n(%)	26(16.05)	12 (30.00)	14 (11.48)	**0.006**
Hyperlipidemia, n(%)	6(3.70)	1 (2.50)	5 (4.10)	0.642
AF,n(%)	65(40.12)	15 (37.50)	50 (40.98)	0.696
Stenosis/occlusion of Intra/extracerebral Arteries,n(%)	96(59.26)	22 (55.00)	74 (60.66)	0.528
**Clinical features**				
GCS, median(IQR)	11(8-13)	11 (7-13)	11 (9-14)	0.157
NIHSS, median(IQR)	16(12-21)	17 (13-21)	16 (12-20)	0.279
Ischemic area≥1/2 MCA territory, median(IQR)	91(56.17)	27 (67.50)	64 (52.46)	0.096
Body temperature,℃ (mean±SD)	36.49±0.37	36.45±0.24	36.50±0.40	0.528
SBP, mmHg (mean±SD)	131.84±1.19	133.84±2.51	131.19±1.35	0.339
DBP, mmHg (mean±SD)	75.60±0.76	76.65±1.63	75.26±0.85	0.430
HR, bpm (mean±SD)	84.48±1.20	89.18±2.65	82.94±1.31	**0.024**
TOAST classification				0.232
large-artery atherosclerosis,n(%)	61(37.65)	16 (40.00)	45 (36.89)	
cardioembolic,n(%)	76(46.91)	21 (52.50)	55 (45.08)	
small-artery occlusion,n(%)	0(0.0)	0(0.0)	0(0.0)	
acute stroke of other determined etiology,n(%)	2(1.23)	1 (2.50)	1 (0.82)	
stroke of underdetermined etiology,n(%)	23(14.20)	2 (5.00)	21 (17.21)	
**In-hospital treatment**				
Thrombolysis, n(%)	26(16.05)	9 (22.50)	17 (13.93)	0.200
Thrombectomy, n(%)	47(29.01)	15 (37.50)	32 (26.23)	0.173
Dehydration	144(88.89)	39 (97.50)	105 (86.07)	**0.046**

therapy,n(%)				
**In-hospital infection**	125 (77.16%)	33 (84.62)	92 (75.41)	0.230

SD, standard deviation; IQR, interquartile range; DM, diabetes mellitus; AF, atrial fibrillation; GCS, Glasgow Coma Scale; NIHSS, National Institutes of Health Stroke Scale; MCA, Middle cerebral artery; TOAST, Trial of Org 10172 in Acute Stroke Treatment.

Table S2 Calculated parameters of overall and epoch-based heart rate-to-blood pressure ratios in patients who died and survived within one month

**Heart rate-to-blood pressure ratio parameters, bpm/mmHg, median (IQR)**	**Total (n=162)**	**One month death (n=40)**	**One month survival (n=122)**	**P values**
**Overall: 1-24h**				
SI_s	0.64(0.56-0.73)	0.67(0.58-0.78)	0.62(0.56-0.72)	0.154
SI_s max	0.85(0.72-1.04)	0.91(0.79-1.10)	0.82(0.72-1.00)	0.037
SI_s min	0.47(0.40-0.54)	0.47(0.39-0.54)	0.47(0.42-0.55)	0.713
SI_s range	0.38(0.26-0.49)	0.67(0.58-0.78)	0.62(0.56-0.72)	**0.022**
SI_s SD	0.10(0.07-0.15)	0.12(0.10-0.16)	0.10(0.07-0.15)	**0.012**
SI_s CV	0.17(0.12-0.22)	0.18(0.15-0.24)	0.16(0.11-0.22)	**0.029**
SI_d	1.14(1.01-1.27)	1.20(1.05-1.36)	1.13(1.01-1.23)	0.074
SI_d max	1.52(1.30-1.79)	1.66(1.39-1.90)	1.50(1.27-1.71)	**0.014**
SI_d min	0.82(0.70-0.92)	0.83(0.69-0.91)	0.82(0.70-0.93)	0.956
SI_d range	0.74(0.49-0.96)	8.35(6.28-11.82)	7.16(4.59-8.86)	**0.007**
SI_d SD	0.20(0.14-0.28)	0.24(0.18-0.30)	0.19(0.13-0.26)	**0.004**
SI_d CV	0.18(0.13-0.23)	0.22(0.16-0.26)	0.17(0.13-0.21)	**0.022**
**Epoch #1: 1-12h**				
SI_s	0.61(0.54-0.71)	0.63(0.55-0.72)	0.60(0.54-0.69)	0.346
SI_s max	0.75(0.65-0.88)	0.81(0.71-0.95)	0.74(0.64-0.86)	0.134
SI_s min	0.48(0.42-0.57)	0.49(0.40-0.59)	0.48(0.43-0.56)	0.654

SI_s range	0.23(0.13-0.38)	2.82(1.85-4.48)	2.00(1.23-3.47)	**0.044**
SI_s SD	0.09(0.06-0.15)	0.12(0.08-0.17)	0.08(0.05-0.13)	**0.029**
SI_s CV	0.15(0.10-0.22)	0.18(0.11-0.26)	0.14(0.09-0.21)	**0.040**
SI_d	1.08(0.94-1.22)	1.11(0.97-1.27)	1.05(0.93-1.21)	0.456
SI_d max	1.32(1.13-1.56)	1.36(1.17-1.61)	1.31(1.13-1.50)	0.229
SI_d min	0.85(0.72-0.95)	0.87(0.68-0.93)	0.83(0.73-0.95)	0.809
SI_d range	0.47(0.27-0.69)	5.38(2.66-7.35)	4.33(2.70-6.47)	0.144
SI_d SD	0.17(0.12-0.26)	0.19(0.13-0.27)	0.16(0.12-0.25)	0.172
SI_d CV	0.28(0.20-0.40)	0.17(0.14-0.24)	0.15(0.11-0.22)	0.140
**Epoch #2: 13-24h**				
SI_s	0.66(0.56-0.77)	0.71(0.61-0.83)	0.64(0.55-0.75)	**0.049**
SI_s max	0.78(0.67-0.93)	0.90(0.77-1.01)	0.75(0.66-0.91)	**0.006**
SI_s min	0.56(0.46-0.64)	0.59(0.47-0.69)	0.55(0.46-0.62)	0.144
SI_s range	0.24(0.15-0.34)	0.28(0.17-0.42)	0.22(0.15-0.33)	0.077
SI_s SD	0.08(0.06-0.13)	0.09(0.06-0.14)	0.08(0.06-0.12)	**0.029**
SI_s CV	0.13(0.10-0.18)	0.14(0.09-0.20)	0.13(0.10-0.17)	0.836
SI_d	1.17(1.04-1.36)	1.26(1.13-1.48)	1.13(1.02-1.29)	**0.013**
SI_d max	1.42(1.21-1.67)	1.56(1.36-1.80)	1.37(1.15-1.65)	**0.004**
SI_d min	0.95(0.83-1.07)	0.97(0.85-1.10)	0.95(0.83-1.06)	0.299
SI_d range	0.48(0.31-0.71)	0.58(0.42-0.79)	0.45(0.30-0.65)	**0.016**
SI_d SD	0.17(0.13-0.25)	0.21(0.15-0.29)	0.16(0.12-0.24)	0.053
SI_d CV	0.15(0.11-0.21)	0.17(0.12-0.23)	0.14(0.11-0.21)	0.289

MBE, malignant brain edema; IQR, interquartile range; SI, shock index; max, maximum;

min, minimum; Heart rate-to-blood pressure ratios are assigned as SI_s and SI_d for systolic and diastolic pressures, respectively; range was defined as the difference between maximum and minimum.
